# Dissociation of the Octameric Enolase from *S. Pyogenes* - One Interface Stabilizes Another

**DOI:** 10.1371/journal.pone.0008810

**Published:** 2010-01-21

**Authors:** Farhad Karbassi, Veronica Quiros, Vijay Pancholi, Mary J. Kornblatt

**Affiliations:** 1 Department of Chemistry and Biochemistry, Concordia University, Montreal, Quebec, Canada; 2 Department of Pathology, Ohio State University, Columbus, Ohio, United States of America; University of Oulu, Finland

## Abstract

Most enolases are homodimers. There are a few that are octamers, with the eight subunits arranged as a tetramer of dimers. These dimers have the same basic fold and same subunit interactions as are found in the dimeric enolases. The dissociation of the octameric enolase from *S. pyogenes* was examined, using NaClO_4_, a weak chaotrope, to perturb the quaternary structure. Dissociation was monitored by sedimentation velocity. NaClO_4_ dissociated the octamer into inactive monomers. There was no indication that dissociation of the octamer into monomers proceeded via formation of significant amounts of dimer or any other intermediate species. Two mutations at the dimer-dimer interface, F137L and E363G, were introduced in order to destabilize the octameric structure. The double mutant was more easily dissociated than was the wild type. Dissociation could also be produced by other salts, including tetramethylammonium chloride (TMACl) or by increasing pH. In all cases, no significant amounts of dimers or other intermediates were formed. Weakening one interface in this protein weakened the other interface as well. Although enolases from most organisms are dimers, the dimeric form of the *S. pyogenes* enzyme appears to be unstable.

## Introduction

**Figure 1:**
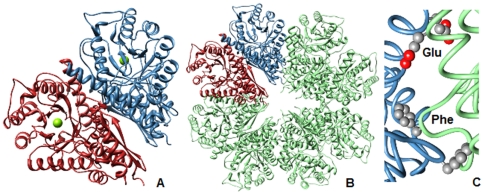
The structure of the octameric enolase of *S. pneumoniae* (1W6T.pdb). A) The dimeric unit showing the monomer-monomer interface, viewed down the β-barrel of the large domain of one subunit. The green ball is the Mg^2+^ at the active site. B) The octamer, with one dimer colored as in Fig. 1A. C) Close up of the dimer-dimer interface, showing F137 (bottom of the figure) and E362 (top pair of residues); residue 362 in *S. pneumoniae* = residue 363 in *S. pyogenes*. One dimer is blue and the other is green, as in Fig. 1B. All figures were made with Chimera (www.cgl.ucsf.edu/chimera/).

Enolase (EC 4.2.1.11) catalyzes the reversible dehydration of 2-phosphoglycerate (PGA) to phosphoenolpyruvate (PEP), an essential reaction in both glycolysis and gluconeogenesis. Depending on the species, enolases have other functions, ranging from being a transcription factor in several organisms [1]–[3] to being a plasminogen receptor on the surface of many cells [4]. Both the primary sequence and the tertiary structure of this protein are strongly conserved. Enolase from most organisms exists as a dimer, with subunit molecular masses of 40–50 kDa. Crystal structures are now available for dimeric enolase from a number of organisms and all show the same basic fold [5]–[8]. The monomer is organized into two domains - a small, N-terminal domain and a larger domain, consisting of an α/β-barrel. The active site is at one end of the barrel with a loop from the small domain contributing to the active site. Subunit contacts are between the small domain of one subunit and the large domain of the other. Octameric enolases have been found in a few organisms [9]–[12]. Electron microscopy of the octameric enolase from *Thermotoga maritima* revealed a ring-shaped structure formed from a tetramer of dimers [11]. This structure has been confirmed by the crystal structure of the octameric enolase from *Streptococcus pneumoniae* [10] (Fig. 1B). The basic fold of its monomer is the same as that in other enolases; formation of the dimer involves the same regions of the two monomers as in the dimeric enolases (Fig. 1A). The dimer-dimer contacts (Fig. 1C) are mainly between identical portions of the two subunits; a region of the small domain contacts the same region in the other subunit, while the end of one helix in the large domain contacts the same region in the other subunit. The N-terminal of each subunit is located towards the center of the ring, while the C-terminal domains form the outer ring.

The active site is completely contained within the monomeric structure, leading to the question: are enolase monomers active? The answer appears to depend on the source of the enolase and the conditions used for dissociation. Dissociation of yeast enolase by chaotropic salts produces monomers that are inactive [13], [14], but have normal secondary structure. Dissociation by dilution and increased temperature produces active monomers [15], [16]. Dissociation by imidazole or low pH produces monomers with low activity [17]. This variability may indicate that the structure necessary for activity is stabilized by subunit interactions and easily lost in their absence.

We are interested in determining the effects of quaternary structure on the function of enolase; we chose to study enolase from *S.pyogenes*. Although a crystal structure is not available for this enolase, it shares 97% homology with that of *S. pneumoniae*, and has been shown, both by mass spectrometry and by modeling, to be octameric [18]. Our goals were to dissociate the octameric enzyme into monomers and dimers in order to compare the octameric, dimeric, and monomeric forms of this enolase. To our surprise, we were not able to produce dimeric enolase.

## Results

### Dissociation and Inactivation by NaClO_4_

**Figure 2:**
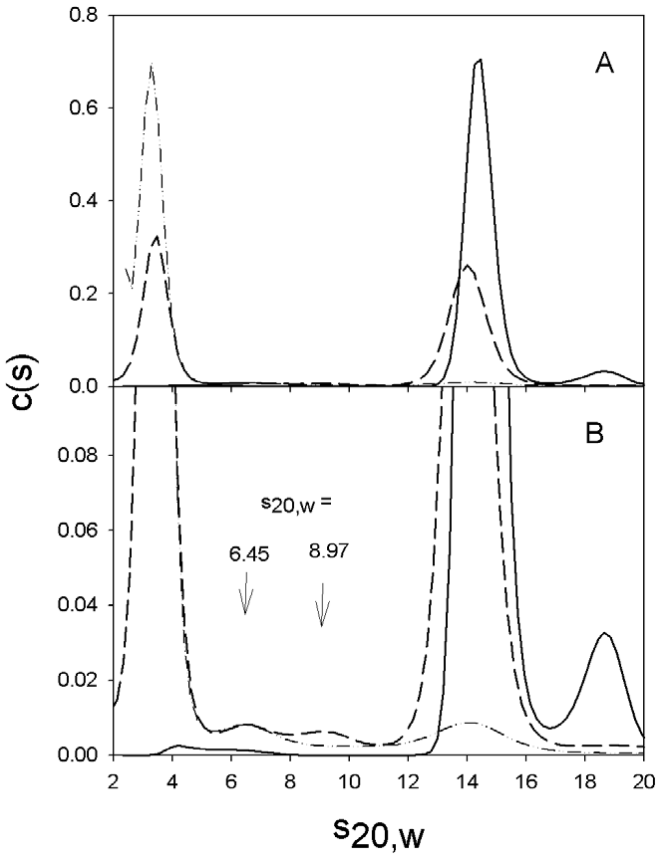
Sedimentation velocity data for the dissociation of the octameric enolase by NaClO_4_. The concentrations of the various species in the sample are shown as a function of their sedimentation coefficients. Prior to centrifugation, the samples were incubated for 18 hours at 15°C in TME (solid line), TME containing 0.2 M NaClO_4_ (long dashes) or TME containing 0.28 M NaClO_4_ (dash-dot-dot). All samples contained 2.7 µM enolase. Centrifugation and data analysis were performed as described in Methods. Fig. 2B is an enlargement of the data shown in Fig. 2A.

**Figure 3:**
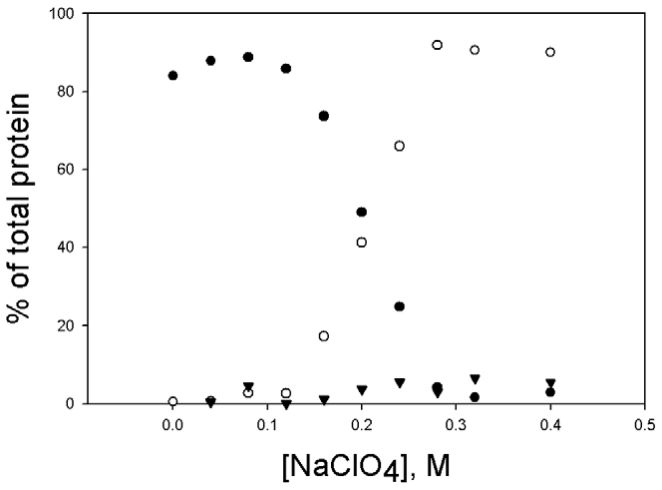
Distribution of species versus concentration of NaClO_4_. Data were obtained as described in Fig. 2. Data for each concentration of NaClO_4_ came from separate incubations and AUC runs, performed over a period of several days. All samples contained 2.7 µM enolase. Solid circles, % octameric; open circles, % monomeric; solid triangles, % all intermediate species.

We have previously shown that NaClO_4_ dissociates yeast enolase into monomers [19]. We therefore incubated the wild type octameric enolase from *S. pyogenes* in varying concentrations of NaClO_4_ and used analytical ultracentrifugation (AUC), in the sedimentation velocity mode, to examine the quaternary structure of the protein (Fig. 2A). In buffer, 84–90% of the protein sedimented with an s_20,w_ of 14.4. Smaller species (1% or less) and larger species (10–15%), including aggregates, were also present. In 0.2 M NaClO_4_, 2 species, with s_20,w_ values of 14.0 and 3.48, were present in almost equal amounts (49% and 41% respectively). As the NaClO_4_ concentration was increased still further, the large peak decreased and the small increased. The major species present in the absence of NaClO4 has an s_20,w_ of 14.48±0.09 (average of three determinations) and a hydrodynamic radius, by DLS, of 6.42±0.04 nm (average of three determinations). The molecular mass calculated from these values is 452±7 kDa, which corresponds to a nonamer. The same AUC data, analyzed by DCDT+ and fitting for s_20,w_ and mass, yields mass values that correspond to septamers. Since it has been sown by mass spectrometry that the enolase from S pyogenes is an octamer [18], we conclude that the major species present in the absence of NaClO_4_, is an octamer. We conclude that the species with s_20,w_ of 3–4 is the monomer, since we have previously shown that the monomer of yeast enolase has an s_20,w_ of 3.35 [19]. The s_20,w_ values for these two species are relatively constant over the range of NaClO_4_ used in these experiments, but they do decrease slightly, which may indicate that changes in tertiary structure are also occurring. Only 4% of the protein was present as intermediate sized species,with s_20,w_ values in the range of 6–9, as can be seen in Fig. 2B. These may correspond to dimers (the dimer of yeast enolase has an s_20,w_ of 5.5 [19]) and/or larger species. The fact that these are present in small amounts and that, in the AUC profiles, there is not complete separation of these species means that there is considerable variation in both the s_20,w_ values and the relative amounts of the various species. By varying the NaClO_4_ from 0 to 0.4 M, we obtained a complete picture of the dissociation process. Fig. 3 shows the concentrations of octamer, monomer and intermediates as a function of the NaClO_4_ concentration.

**Figure 4:**
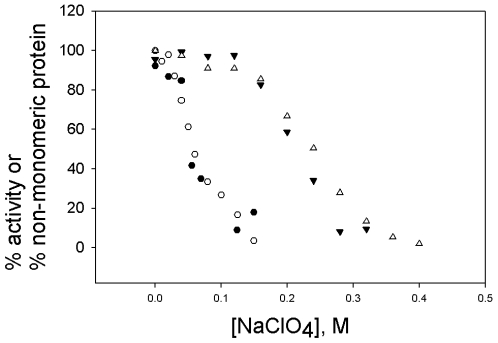
Dissociation and inactivation of the wild type octameric enolase and the F137L/E363G variant by NaClO_4_. Enolase, 2.7 µM was incubated in NaClO_4_ as described in Figs. 2 and 3. Prior to loading the AUC cells, each sample was assayed for enzymatic activity. Open symbols, % activity; closed symbols, % non-monomeric protein (calculated at 100% - % monomeric); circles, F137L/E363G variant; triangles, wild type enolase.

Incubation in NaClO_4_ also inactivated the enzyme. This loss of activity correlated closely with formation of monomers (Fig. 4), suggesting that all of the various species, with the exception of monomers, were active.

### Effects of Mutations at the Dimer-Dimer Interface

**Figure 5:**
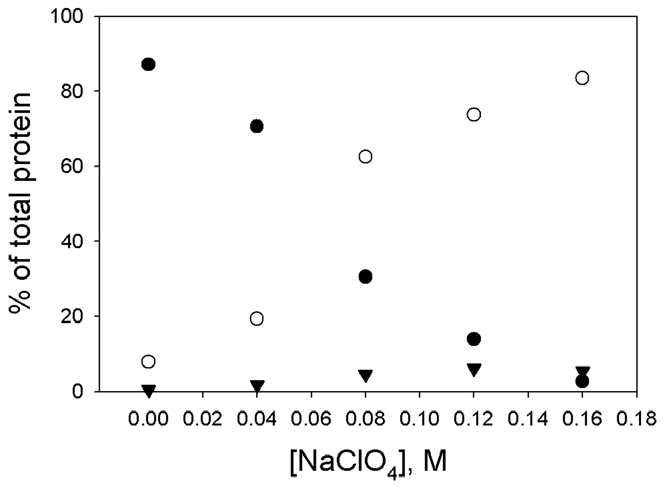
Distribution of species versus concentration of NaClO_4_ for the F137L/E363G variant. Data were obtained from experiments performed as described in Figs. 2 and 3. All samples contained 2.7 µM enolase. Solid circles, % octameric; open circles, % monomeric; solid triangles, % all intermediate species.

Since one of our original objectives was to study the dimeric form of the *S. pyogenes* enolase, we studied a variant that had two mutations, F137L and E363G, at the dimer-dimer interface (Fig. 1C). Our assumption was that these mutations would weaken the dimer-dimer interface, but have no effect on the monomer-monomer interface. This variant was expressed, and purified. Based on the circular dichroism (CD) spectrum in the peptide bond region, the double mutant was correctly folded; the specific activity of the double mutant in our standard enzymatic assay was 80–85% that of the wild type enzyme. The effects of NaClO_4_ on the activity and quaternary structure of this variant were then studied. The changes at these two positions destabilized the structure of the protein. 50% dissociation and inactivation occurred at about 0.06 M NaClO_4_, as opposed to 0.2 M for the wild type protein (Fig. 4). As with the wild type enzyme, dissociation of the octamer into monomers was accompanied by formation of only small amounts of intermediate species (Fig. 5). The Tm for denaturation for this variant, monitored by CD at 220 nm, was decreased, from 68°C for the wild type to 61°C.

Both dissociation and inactivation of the variant form were largely, but not completely, reversible. Enolase, at 19.4 µM, after incubation in 0.2 M NaClO_4_, had 32% of its original activity and was 21% octameric. Following a 24-hour dialysis against buffer, the protein was 77% octameric and had 74% of its original activity.

### Questions and Other Experimental Approaches

**Figure 6:**
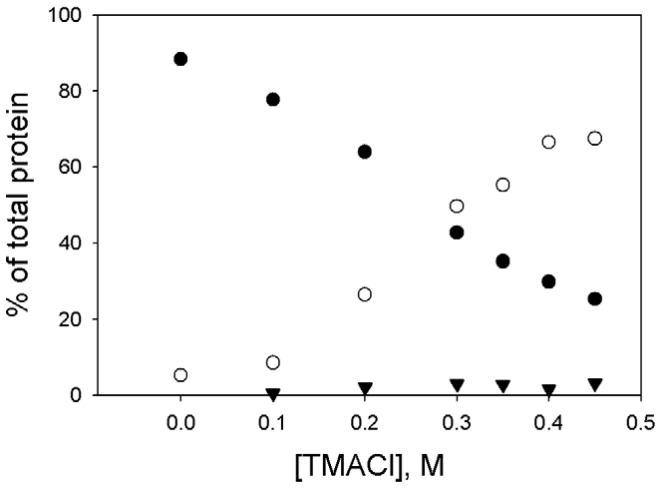
Distribution of species versus concentration of TMACl for the F137/E363G variant at 2.7 µM. Data were obtained from experiments performed as described in Figs. 2 and 3. All samples contained 2.7 µM enolase. Solid circles, % octameric; open circles, % monomeric; solid triangles, % all intermediate species.

Are these results due to the perchlorate ion acting as an oxidizing agent? The fact that both dissociation and inactivation are largely reversible means that neither process is due to irreversible covalent modifications of the protein, such as oxidation. Are these results due to the presence of Na^+^, a known inhibitor of enolase [20]? In order to answer this question, the ability of other salts to dissociate the octameric enolase was examined. These experiments were all performed using the F137L/E363G variant. The effects of TMACl were qualitatively the same as those of NaClO_4_; the octameric enolase was dissociated into monomers (Fig. 6); the maximum concentration of intermediates was 3%. Although not studied in detail, NaCl and KCl had the same effects as TMACl. In 0.4 M NaCl or KCl the enzyme was 70–80% monomeric. All three salts also inactivated the enzyme. However, 0.4 M NaOAc neither dissociated nor inactivated the enzyme. These results establish that dissociation of the octameric enolase into monomers is not a specific effect of either ClO4^−^ or Na^−^. Dissociation is produced by salts of chaotropic ions [21], such as ClO4^−^ and Cl^−^, but not by salts of kosmotropic ions such as Oac^−^.

Dissociation also occurred if the pH was increased. At pH 7.4, the variant enzyme was 88% octameric; at pH 9.0, it was 39% octameric, with 2% intermediates and 54% monomers. By pH 10 in glycine buffer, it was 88% monomeric; less than 1% was present as intermediate species. As with salts, this dissociation was not accompanied by formation of intermediates. High pH also inactivated the enzyme.

**Figure 7:**
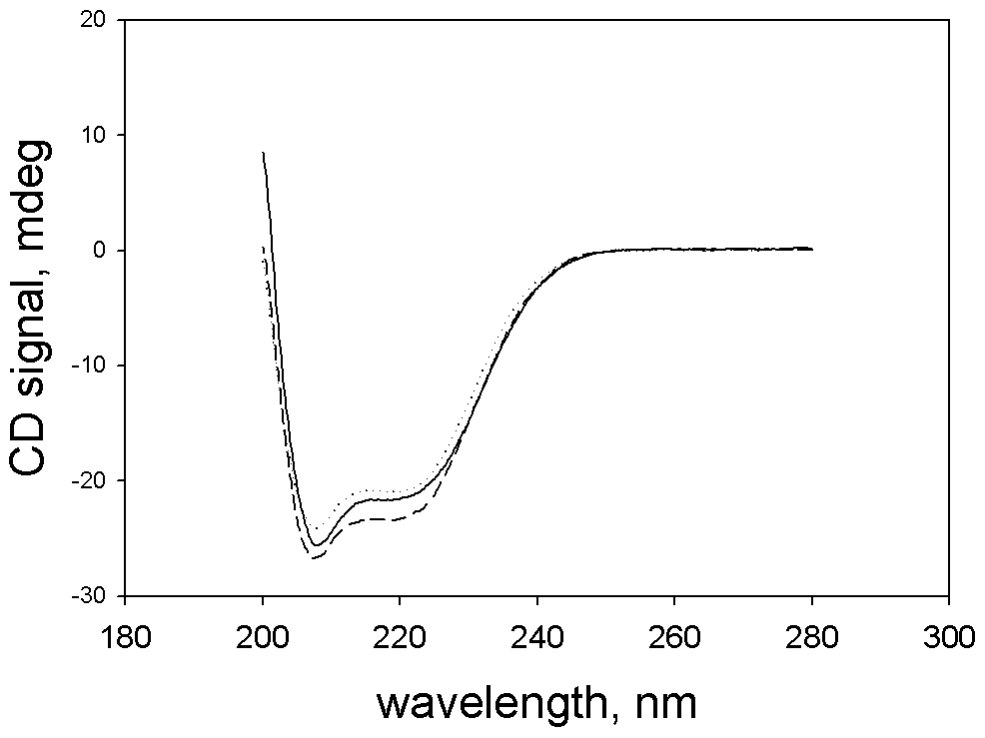
CD spectra of the variant enolase in its octameric and monomeric forms. The concentration of enolase in all samples was 4.1 µM. Samples were incubated for 18 hours at 20°C; the CD spectra were then recorded as described in Methods. Solid line, enzyme in TME; dotted line, enzyme in TME plus 0.2 M NaClO_4_; dashed line, enzyme in glycine buffer, pH 10.

Does dissociation involve denaturation? Dissociation, whether by NaClO_4_ or by high pH, was not accompanied by loss of secondary structure, as determined by peptide bond circular dichroism (CD) (Fig. 7). (In contrast, complete dissociation of the wild type enzyme, which requires 0.4 M NaClO_4_, was accompanied by partial unfolding.)

### Time Course of Dissociation and Inactivation

**Figure 8:**
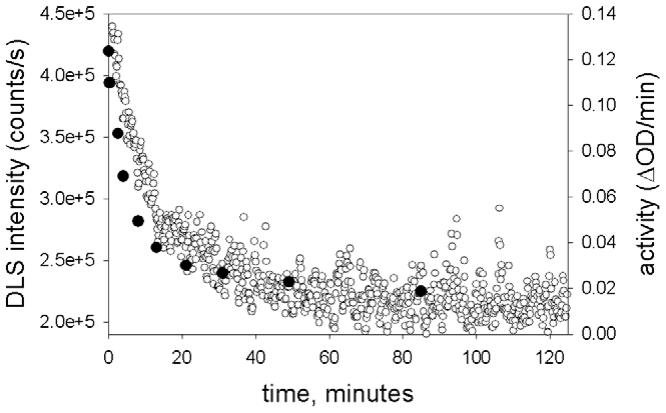
Time course of changes in DLS intensity and activity during incubation in NaClO_4_. NaClO_4_ was added to a solution of 2.7 µM enolase; the sample was immediately placed in the DLS cuvette and measurements begun or aliquots were taken at various times for activity measurements. For both DLS and activity measurements, the sample was held at 15°C. Open circles, DLS intensity; solid circles, activity.

The above experiments were all performed under equilibrium conditions. In order to examine more closely the correlation between dissociation and inactivation and to look for evidence of a step-wise process, we wished to follow the time course of both processes. In a dynamic light scattering (DLS) experiment, the intensity of the signal from large molecules is greater than that from small molecules. Dissociation of the octameric enzyme will result in a decrease in the intensity of the signal. NaClO_4_ was added to a sample of enzyme and the DLS signal was monitored as a function of time. In a parallel experiment, NaClO_4_ was added to enzyme, and aliquots were removed and assayed at various times. As can be seen in Fig. 8, the decrease in DLS signal and the loss of activity followed the same time course; in addition, there was no indication that multiple processes were occurring.

## Discussion

Sedimentation velocity experiments clearly showed that incubation in NaClO_4_ produced changes in the quaternary structure of *S. pyogenes* enolase; the octameric enzyme was dissociated into monomers that were inactive. We have shown that both the inactivation and dissociation are reversible. However, the fact that we observe discrete species in the AUC experiments indicates that equilibration among the species is slow.

The time course of dissociation, monitored by DLS, and the time course of inactivation are very similar. Both sets of data (inactivation vs. time and DLS intensity vs. time) in Fig. 8 can be fit to an equation for single exponential decay (fit not shown). There is no indication in this data of the occurrence of multiple steps or the presence of significant amounts of intermediates.

The octameric protein dissociates into monomers, without the formation of large amounts of dimers. In the various dissociation experiments that we performed, the maximum concentration of the intermediate species was less than 10% of the total protein and was observed when dissociation was almost complete (Fig. 3 and 5). This is not the behavior one would expect of a true intermediate and may indicate that these intermediates are formed from the association of monomers. The fact that there is no intermediate whose concentration builds up and then decreases suggests either of two pathways: 1) the octamer dissociates directly into monomers or 2) the octamer dissociates into some smaller species which then rapidly dissociates into monomers. Both dissociation and inactivation have a dependence on protein concentration. With the variant enzyme, the concentration of NaClO_4_ necessary for 50% dissociation is 0.06 M when the protein concentration is 2.7 µM but increases to 0.12 M when the protein concentration is 22.7 µM. However, this concentration dependence is not great enough to be due to an octamer dissociating into 4 dimers or 8 monomers, but could be explained by an octamer dissociating into two tetramers.

We have previously shown that yeast enolase is dissociated by NaClO_4_; under the same concentrations used in these experiments, complete dissociation of the yeast enzyme requires 0.3 M NaClO_4_ [19]. The monomer-monomer interface of the *S. pneumoniae* enolase is similar to that of other enolases in the amount of buried surface area; groups contributing hydrogen bonds or electrostatic interactions in the yeast dimer interface [5] are also present in the *S. pyogenes and S. pneumoniae* enolases. The two interfaces of the octameric enolase from *S. pneumoniae* are similar in amount of buried surface and the ratio of charged to hydrophobic residues (1.1 for the monomer-monomer interface and 0.8 for the dimer-dimer interface [10].) Although NaClO_4_ is a chaotropic salt, it will also weaken electrostatic interactions. Thus the fact that complete dissociation of the *S. pyogenes* enolase into monomers also occurred at 0.3 M NaClO_4_ was not surprising. What was surprising were the results with the F137L/E363G variant. We fully expected that this variant would be dissociated into dimers, since both changes are at the dimer-dimer interface (Fig. 1C). This variant was more easily dissociated than was the wild type, but did not dissociate into dimers. These changes have destabilized **both** the dimer-dimer and the monomer-monomer interface. The dimer-dimer interface stabilizes the monomer-monomer interface, in effect stabilizing the active, functional unit.

Are these results specific to NaClO_4_? We have shown that dissociation can also be produced by incubation of the enzyme with NaCl, KCl, and TMACl, but not with NaOAc. Dissociation by salts was not an effect of Na^+^ or K^+^ ions, nor of high ionic strength; it did require a chaotropic salt. Dissociation could also be produced by alkaline pH. In all of these cases, the characteristics of the dissociation are the same – the octamer dissociated into monomers with little formation of dimers or other intermediates, the monomers were inactive, and there was no loss of secondary structure.

The vast majority of enolases are dimeric, with the same monomer-monomer interface that is seen in the octameric enolases. Yeast enolase is fully dimeric with K_diss_ = 1.5×10^-8^ M [19]. Enolases from a small number of organisms are octameric, with the eight subunits arranged as a tetramer of dimers, forming a ring (Fig. 1A). There are other enzymes which, depending on source, occur in multiple quaternary states. Cytosolic creatine kinases are dimers, while the mitochondrial creatine kinase is an octamer [22] formed by 4 dimers [23]. Phosphoglycerate mutase from vertebrates is dimeric while the enzyme from yeast is tetrameric [24], and dihydrodipicolinate synthase can be either tetrameric or dimeric [25]. In all of these cases, the larger form of the enzyme can be dissociated into dimers, either by changing conditions or by introducing mutations. Arginine decarboxylase of *E. coli* exists as either a decamer or as a dimer, with the equilibrium between the two forms shifted by pH [26]. Formiminotransferase-cyclodeaminase is also an octamer, with the subunits arranged in a ring as a tetramer of dimers [27]. This protein can be dissociated by urea into dimers; depending upon conditions during the urea treatment, either subunit interface can be disrupted producing either of two types of dimers [28].

In these examples, the two types of interfaces present in the oligomer appear to be independent - one can be disrupted while maintaining the other. This is not the case for enolase from *S. pyogenes*. When we weakened one set of interactions by mutations we weakened the other set as well; there is cooperativity between the two interfaces.

With the octameric enolase, there are differences between the octamer and monomer in the aromatic CD spectrum, fluorescence and binding of 8-anilino-1-napthalene sulfonic acid (data not shown). We have previously shown that dissociation of yeast enolase produced subtle changes in the structure of the monomer; there was a small increase in disordered structure and several peptide bonds, including one in a helix far from the interface, became susceptible to proteolysis [19]. However, none of these observations shed light on the nature of the communication between the two interfaces. The monomer-monomer interface in *S. pyogenes* enolase involves the small domain of one subunit and the large domain of the other. The dimer-dimer interaction mainly involves small domain with small domain. Communication between the two interfaces could be via the two faces of the small domain and/or via the orientation of the large domain relative to the small.

Enolases have not been obvious drug targets, since enolase is an essential enzyme and all enolases studied to date use the same mechanism. Recently, there have been suggestions that small structural differences between enolases could be exploited to selectively inhibit enolases from parasitic protozoa [6], [29]. In addition to its essential role in glycolysis, enolases from many pathogenic bacteria are also plasminogen receptors [30]–[32]. To date, there is little information about the relationship between enolase structure and plasminogen binding, although one mutation that partially dissociates enolase appears to increase binding [18]. Compounds designed to disrupt the dimer-dimer interface of the *S. pyogenes* enolase should have no effect on the dimeric mammalian enolases. The potential of such compounds to affect infectivity or plasminogen binding are worth examining.

## Methods

### Mutagenesis, Expression and Purification

The gene for enolase from *S. pyogenes* had previously been cloned and inserted into the pET-14b plasmid [33]; the resulting plasmid codes for a protein with an N-terminal his-tag. *E. coli* strains XL1Blue and BL21(DE3) were used for storage and expression of the plasmid respectively. The protein coded for by the initial plasmid differed from the wild type protein at two positions: 137 and 363. These two positions were changed to the wild type positions by mutagenesis, using the QuickChange method (Stratagene, La Jolla, CA, USA) and oligonucleotides from *Mtl*Biotech (DDO, Quebec, Canada). Enzymes for molecular biology were from Fermentas Canada (Burlington, Ont. Canada). The presence of the desired mutations was verified by sequencing the gene (BioS&T, Inc., Lachine, Canada) and by Q-ToF mass spectrometry (CBAMS, Concordia University, Canada). Protein with the correct sequence is referred to as wild type enolase; protein expressed from the original plasmid is referred to as the F137L/E363G variant.

E coli, BL21(DE3), containing the desired plasmid, was grown in Luria broth containing ampicillin (BioShop Canada, Inc, Burlington, Ont. Canada) at 37°C until an OD_650_ of 0.6 was reached. Isopropylthiogalactopyranoside (BioShop Canada Inc.) was added to a final concentration of 0.5 mM, the temperature was lowered to 29°C and the cells grown for an additional 4 hours. The cells were harvested by centrifugation and stored at −20°C. The cells were thawed, suspended in washing buffer (20 mM HEPES, 300 mM NaCl, 10 mM imidazole, 1 mM Mg(OAc)_2_, pH 7.5) containing 0.06 mg ml^−1^ of DNAse and 0.06 mg ml^−1^ of RNAse and sonicated. The crude extract was centrifuged and loaded onto a Ni-NTA column (Qiagen, Inc, Mississauga, Ont., Canada). Following washing, the protein was eluted with elution buffer (20 mM HEPES, 300 mM NaCl, 250 mM imidazole, 1 mM Mg(OAc)_2_, pH 7.5). The protein was immediately dialyzed overnight against a 100-fold excess of TME buffer (50 mM Tris/HCl, 1 mM Mg(OAc)_2_, 0.1 mM EDTA, pH 7.4). The protein was then precipitated by 85% (NH4)_2_SO_4_ and stored at 4°C as the precipitate. Prior to use in any experiments, the enzyme was resuspended and dialyzed against TME buffer. All of the above steps were performed at 4°C.

### Enzymatic Assays

The enzyme activity was monitored by following the conversion of PGA to PEP at 240 nm The assay buffer contained 50 mM HEPES, pH 7.5, 1 mM Mg(OAc)_2_, and 1 mM PGA; assays were performed at room temperature. Since Na^+^ inhibits other enolases [20], the HEPES was titrated with KOH. Activity measurements are the average of duplicates, except for the experiment in Fig. 5. The concentration of enolase was determined from the absorbance at 280 nm and expressed as concentration of subunits. Extinction coefficients were calculated from the amino acid composition [34]. For the wild type enzyme and the double mutant, ε = 43320 M^−1^ cm^−1^. PGA was synthesized enzymatically from ATP and glyceric acid [35].

### Dissociation of Enolase

Enolase was incubated in TME buffer containing varying concentrations of NaClO_4_ or other salts for 18–24 hours at 15°C; the activity of the enolase was measured and the samples were used for the various biophysical measurements. When pH was varied, 50 mM Tris or Glycine, containing 1 mM Mg(Oac)_2_ and 0.1 mM EDTA, was adjusted to the desired pH at 20°C. Incubations of the enzyme in these buffers were also at 20°C. The concentration of enolase in most experiments was either 2.7 µM monomers or 22.7 µM monomers. For experiments monitoring the time course of changes, the measurements of activity and light scattering were begun within 1 minute of adding NaClO_4_ to the sample.

### Analytical Ultracentrifugation

Sedimentation velocity experiments were performed in a Beckman (Fullerton, CA, USA) XL-I analytical ultracentrifuge at the Concordia University Center for Structural and Functional Genomics. Samples were centrifuged at 35,000 rpm in a Ti60 rotor for 12–16 hours at 15°C, except for the pH studies which were performed at 20°C, and monitored at either 230 nm or 280 nm. Data were analyzed using SEDFIT89 [36] (available at www.analyticalultracentrifugation.com), using the continuous c(s) distribution model. To determine the mass of the undissociated species, a few AUC runs were also analyzed by DCDT+,version 2.02 (available from J. Philo at www.jphilo.mailway.com), fitting for s_20,w_ and mass. The partial specific volume of the protein and the viscosity and density for each buffer were calculated using SEDNTERP, version 1.07 (D.B. Hayes, T. Laue and J. Philo, available at www.bbri.org/RASMB/rasmb.html) and used to convert sedimentation coefficients to s_20,w_.

### Circular Dichroism and Dynamic Light Scattering

Circular dichroism spectra were recorded on a Jasco J-815 spectropolarimeter, with a thermostatically controlled sample compartment. The concentration of these protein was 4.1 µM. Samples, at 20°C, were scanned from 260 to 200 nm at 20 nm per second with a 1 nm bandwidth and a 1 second response time. Five spectra were recorded and averaged. Spectra of the corresponding buffers were recorded using the same parameters. The Jasco software was used for subtraction of the buffer spectra and smoothing. For temperature denaturation studies, the samples were monitored at 220 nm over a temperature range of 35° to 80°C at a rate of 15°C per hour.

DLS measurements were made on a Dyna Pro instrument (Wyatt Technology, Santa Barbara, CA, USA), with the cell compartment thermostated at 15°C. Protein concentration was 2.8 µM. 10-second acquisitions, with a laser power of 100%, were recorded continuously for 6 hrs.
